# Healthy lifestyle over the life course: Population trends and individual changes over 30 years of the Doetinchem Cohort Study

**DOI:** 10.3389/fpubh.2022.966155

**Published:** 2022-09-09

**Authors:** Edith E. Schermer, Peter M. Engelfriet, Anneke Blokstra, W. M. Monique Verschuren, H. Susan J. Picavet

**Affiliations:** ^1^Center for Nutrition, Prevention and Health Services, National Institute for Public Health and the Environment, Bilthoven, Netherlands; ^2^Julius Centre for Health Sciences and Primary Care, University Medical Centre, Utrecht, Netherlands

**Keywords:** lifestyle and behavior, cohort, trends, overweight and obesity, physical activity, sleep, alcohol consumption, smoking-epidemiology

## Abstract

For five health-related lifestyle factors (physical activity, weight, smoking, sleep, and alcohol consumption) we describe both population trends and individual changes over a period of 30 years in the same adult population. Dichotomous indicators (healthy/unhealthy) of lifestyle were analyzed for 3,139 participants measured every 5 years in the Doetinchem Cohort Study (1987–2017). Population trends over 30 years in physical inactivity and “unhealthy” alcohol consumption were flat (i.e., stable); overweight and unhealthy sleep prevalence increased; smoking prevalence decreased. The proportion of the population being healthy on all five lifestyle factors declined from 17% in the round 1 to 10.8% in round 6. Underlying these trends a dynamic pattern of changes at the individual level was seen: sleep duration and physical activity level changed in almost half of the individuals; Body Mass Index (BMI) and alcohol consumption in one-third; smoking in one-fourth. Population trends don't give insight into change at the individual level. In order to be able to gauge the potential for change of health-related lifestyle, it is important to take changes at the individual level into account.

## Introduction

For public health policy it is important to monitor health and health determinants in the population. This allows discerning worrisome trends in health determinants that require attention, such as an increasing prevalence of obesity, or evaluating the effects of interventions such as anti-smoking campaigns. In addition to these population trends it is relevant to know what the dynamics are that drive these trends. Trends over time in a population are the “net sum” of all changes in individuals who make up that population; visualizing the latter shows a much more nuanced picture than tracing the population average. To take an extreme example: a flat (unchanging) trend in average BMI could mean that most individuals maintain a stable weight, or in contrast that half of the population loses weight while the other half gains weight. This might lead to very different conclusions regarding the need (and potential) for policy interventions. To bring to light the spectrum of changes at the individual level, it is necessary to follow the individuals making up the population over time and summarize these changes in an insightful manner.

Health-related lifestyle factors that are often studied are: physical activity, overweight, smoking, sleep, and alcohol consumption, for which there is substantial knowledge about the population trends (1–5). In general, in high-income countries, levels of physical activity are relatively stable over time (1), the prevalence of being overweight is increasing (2), while smoking prevalence is decreasing (3). There is inconclusive evidence on trends in sleep duration; whereas some countries show an increase in average sleep duration of its inhabitants, others in contrast show a decrease (4). Similarly, per capita average daily alcohol consumption decreased in some high-income countries and remained stable in others (5).

There is increasing interest in tracking individual changes in lifestyles using longitudinal cohort data (6, 7). These individual changes, often referred to as trajectories, can be described using predefined categories, or evaluated using data-driven methods. For example, trajectories identified for physical activity can be described as moderately stable or highly stable, or in contrast as showing steep or steady in- or decreases over time (8, 9). Trajectories of weight are often identified for specific age groups, like old age (10), or over transition periods, like from adolescence into adulthood (11). An example of an often observed trajectory of alcohol consumption throughout adult life is one that shows alcohol consumption first rising, then reaching a plateau, and finally declining in older age (12, 13). As another example, a 46-year follow-up study found that smoking behavior remained stable in over 60% of its adult population (14). We have previously shown that half of the adults aged 20 years and above remain stable in their physical activity level and sleep duration over a period of 20 years (15, 16).

In addition to studying single lifestyle factors, it is relevant to study combinations of lifestyle factors in order to be able to anticipate the increase in morbidity and mortality in the population due to the accumulation of unhealthy habits (17). Papers studying several health-related lifestyle factors simultaneously over time are scarce.

Using 30 years of longitudinal data from the Doetinchem Cohort Study, we aim to analyze both population and individual level changes within the same study population. For the population level the time trends refer to circa 30 years and for the individual level the data spans over circa 25 years. In particular, we focus on physical activity, Body Mass Index (BMI), smoking, alcohol consumption, and sleep. The leading question is: what are the population trends *and* individual changes for five health-related lifestyle factors (physical activity, weight, smoking, sleep, and alcohol consumption)—separately *and* in combination—in the same adult population?

## Methods

### Design, study population, and data collection

**Figure 1:**
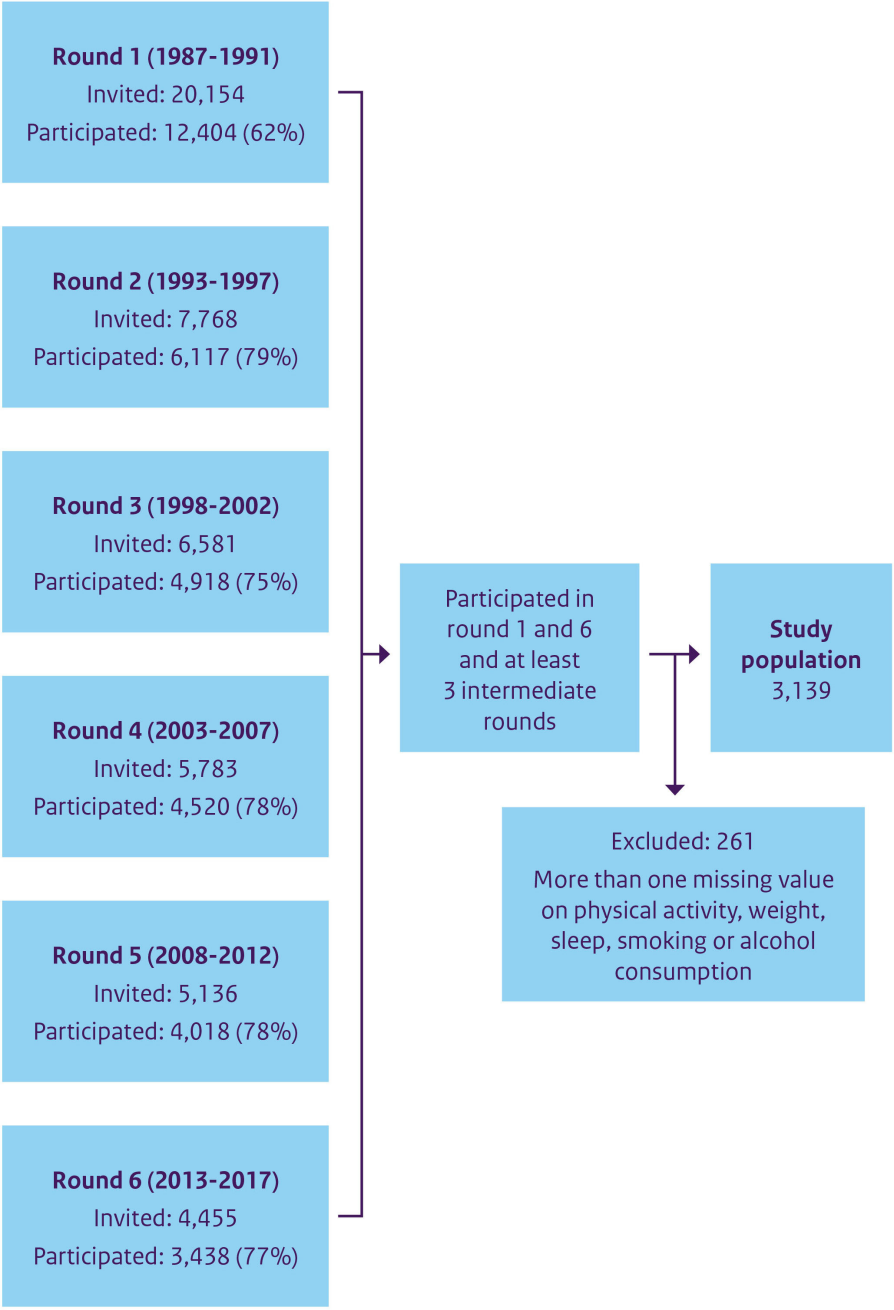
Flowchart of the participants of the Doetinchem Cohort Study from round 1 till round 6.

Data was used from the Doetinchem Cohort Study (DCS), a prospective population-based cohort study in a midsize (provincial) town in the Eastern part of the Netherlands. Starting in 1987 as a monitoring study on risk factors for cardiovascular disease, the DCS continues to this day. The study population consists of an age-sex-stratified random sample of the Doetinchem mainly white (Caucasian) population, measured every 5 years in measurement rounds that take 5 years to complete. In the first round, 12,404 individuals participated, more than half of whom were subsequently invited to participate in the following examination rounds (18). The flowchart (Figure 1) shows the participants in each round. By using questionnaires and a physical examination, data on lifestyle and health is collected. In this study, data were included from round one (1987–1991) through round six (2013–2017), covering a 30-years period of data collection that allow tracking individual changes over a time span of 25 years. Participants were included in the analyses if they took part in the first and sixth rounds and at least three intermediate rounds.

### Lifestyle factors

We included physical activity, overweight, smoking, sleep, and alcohol consumption because we had comparable data on indicators of these factors over the complete 30 year-period in the same cohort. Each lifestyle factor was dichotomized as “healthy” or “unhealthy”, based on guidelines and literature, so we could use the same analyses for every indicator. Moreover, in monitoring of population trends, variables of interest such as BMI are also often dichotomized (yes/no overweight), in order to define a proportion of the population at increased risk based on well-motivated cut-off values.

Physical activity was assessed with a questionnaire from the European Prospective Investigation in Cancer and Nutrition study (EPIC) (19). Adherence to the Dutch physical activity guidelines (20)—being being at least moderately active for 30 min per day, for at least 5 days per week—was used as criterion to define participants as active or inactive, which corresponds to 150 min per week. Due to the extensive nature of the EPIC questionnaire with overlapping categories, overreporting of physical activity was taken into account and corrected for, similarly to previous studies using the same cohort (15, 21), by using 210 min of at least moderate activity per week as cut-off point. Body Mass Index (BMI), derived from individuals' measured height (m) and weight (kg), was used to define overweight (BMI of ≥ 25 kg/m^2^, i.e., unhealthy). For smoking, non-smokers and former smokers were classified as healthy, current smokers as unhealthy. For sleep, sleep duration was assessed with one self-reported question. The recommended sleep duration for adults is 7–8 h (22). Participants were defined as unhealthy if they slept <7 h or more than 9 h. For alcohol consumption, the number of glasses per day was used. Based on guidelines by the Dutch health Council in 2015, consuming more than one (>1) glass per day was classified as unhealthy. The numbers of missings were low and varied per lifestyle factor. For the analyses per lifestyle factor those with missings were excluded, so the actual numbers slightly differed per analysis. Those with missings on e.g., smoking were not excluded from the analyses of physical activity.

### Sociodemographic characteristics

We were also interested in differences between groups, defined according to sex, age (as generation-definition), and level of education. Sex assigned at birth was used (as registered in the population registers). Four age groups were created based on age at the baseline measurement and labeled as “10-year generations”: those in their 20s- 30s-, 40s- or 50s at baseline. Over the course of the study, all participants became 25 years older, e.g., those who were in their 40s- at baseline were between 65 and 74 years old in the sixth measurement round. Educational level was based on questionnaire data on highest attained level until the fourth examination round, and categorized into three levels: low, intermediate, and high. Low level meant intermediate secondary education or less, intermediate level was intermediate vocational and higher secondary education, and high level meant higher vocational education and university.

### Statistical analyses

For the, mainly descriptive, statistical analyses, SAS version 9.4 was used. Population trends were determined by estimating the prevalence of the unhealthy lifestyle factor in each of the consecutive rounds. Individual changes were determined by utilizing the classification of the lifestyle factors into healthy (*h*) or unhealthy (u) Participants had a maximum of six measurements at different time points, resulting in 64 (2^6^) possible patterns of healthy (h) and unhealthy (u) (not counting missing values). These were grouped into five different patterns. Respondents could remain “stable healthy” or “stable unhealthy” or change in a healthy (“improve”), or unhealthy direction (“worsen”), or “vary” over time. If there was only one intermediate deviating value, or a missing value, the participants were categorized based on the other measurements. For instance, participants with the pattern *hhhuhh* or *uhuuuu* would be categorized as respectively “stable healthy” or “stable unhealthy” on that factor. To be classified as “varying” over time, participants had to vary between healthy and unhealthy more than once (e.g., *uhuhuh*): those were the participants that could not be classified into one of the other patterns.

For each lifestyle variable the distribution of the population over these five patterns was determined and expressed as proportions of the population adhering to these patterns.

Besides the trends and individual changes of the single lifestyles we present the proportion of participants that were healthy or unhealthy on *all* of the studied lifestyle factors and for how many of the five lifestyle factors individuals changed during the study. Participants that either “improved”, “worsened”, or “varied” in a lifestyle factor were classified as having changed that factor, resulting in the possibilities to have changed zero to five factors. Remaining stable in all five factors was labeled as being “stable healthy” or “stable unhealthy”.

## Results

A total of 3,139 participants were included in the study, 53% of whom were women (Table 1). The average age at baseline was 37.6 years and 63.6 years in the sixth round. Most participants had a low educational level (42.2%), followed by intermediate (31.2%) and high educational level (26.5%).

**Table 1:** Characteristic of the study population (*n* = 3,139).

**Sex**	***N***	***%***		
Men	1,475	47.0		
Women	1,664	53.0		
**Average age (years)**	**mean**	**sd**		
Baseline–Round 1 (1987–1992)	37.6	9.2		
Round 2 (1993–1997)	43.7	9.2		
Round 3 (1998–2002)	48.6	9.2		
Round 4 (2003–2007)	53.6	9.2		
Round 5 (2008–2012)	58.5	9.2		
Round 6 (2012–2017)	63.6	9.2		
**10-year generations, based on baseline age**	***N***	**%**		
20s (20–29 years)	647	20.6		
30s (30–39 years)	1,155	36.8		
40s (40–49 years)	955	30.4		
50s (50–59 years)	382	12.2		
**Average age per round per 10-year generation (years)**	**20s**	**30s**	**40s**	**50s**
Round 1 (1987–1992)	25.0	34.4	43.6	53.3
Round 2 (1993–1997)	31.1	40.4	49.6	59.3
Round 3 (1998–2002)	36.0	45.4	54.6	64.2
Round 4 (2003–2007)	41.0	50.4	59.6	69.2
Round 5 (2008–2012)	46.0	55.4	64.5	74.1
Round 6 (2012–2017)	51.0	60.5	69.6	79.2
**Educational level**	***N***	**%**	
Low	1307	42.2		
Intermediate	966	31.2		
High	821	26.5		

### Population trends of single health-related lifestyle factors over 30 years

**Figure 2:**
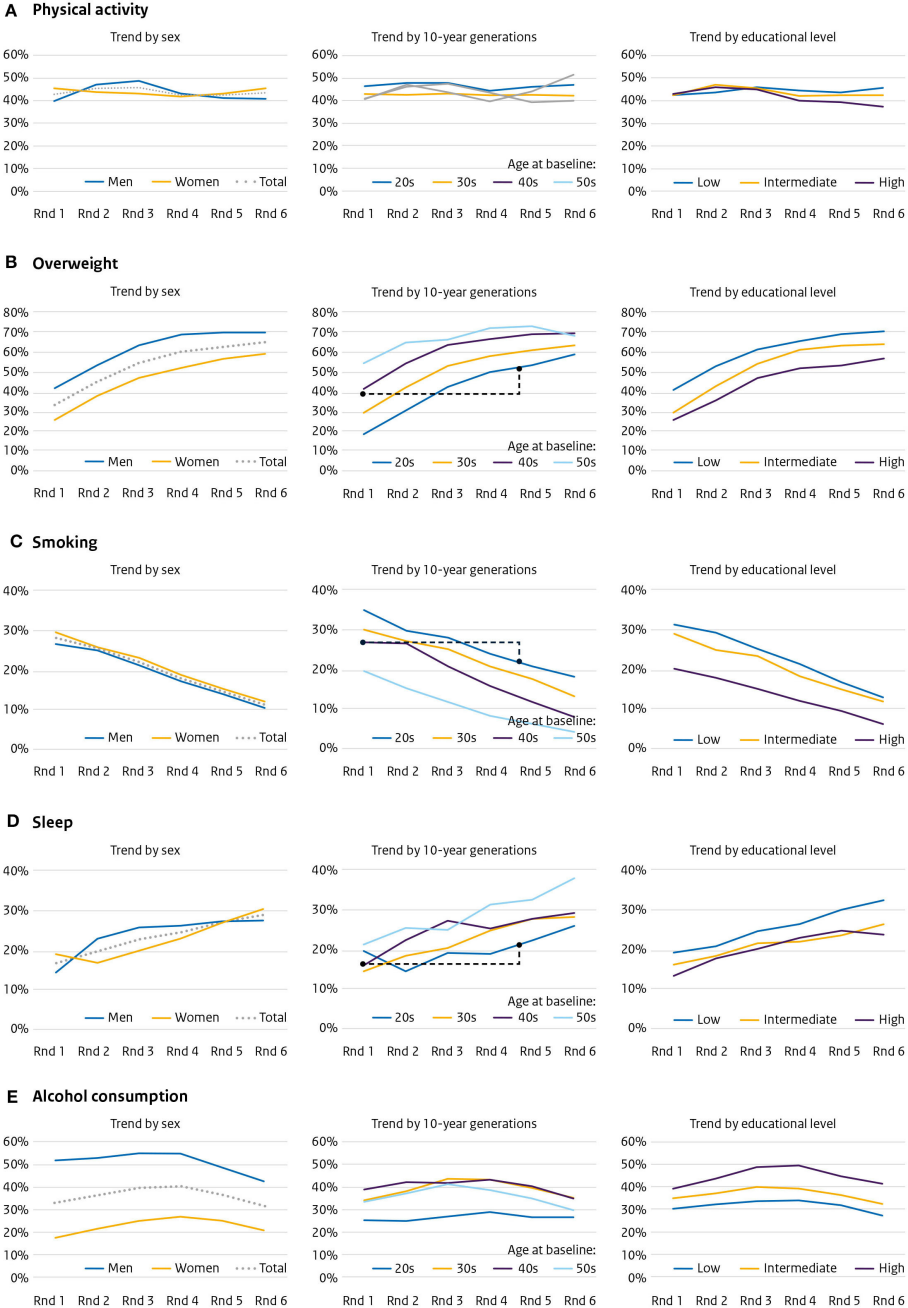
Population trends over 30 years—six measurement rounds with a duration of 5 years (1987–2017)—for **(A)** physical activity, **(B)** overweight, **(C)** smoking, **(D)** sleep, and **(E)** alcohol consumption, by sex, 10-year generation and educational level.

The proportion of adults not meeting the norm for physical activity remained relatively stable during the 30 years of the study: ~43% (Figure 2A). No notable differences were found between the 10-year generations or the different educational levels. The proportion of overweight participants (BMI ≥25 kg/m^2^) increased from 41.9 to 69.9% for men and from 26.6 to 58.9% for women (Figure 2B). All 10-year generations showed an increase, and every younger 10-year generation had a bigger proportion of overweight participants than their predecessors: e.g., those in their 20s at baseline were more often overweight by the age of 40 (52.5%) than those in their 40s at baseline (42.3%) (indicated by the dotted black line). During the entire study period, low educated participants were more often overweight (from 40.9 to 69.1%) than high educated participants of the same age (26.1 to 56.4%). In both men and women, smoking prevalence declined, on average, from 28.2 to 11.4% (Figure 2C). Participants in their 20s at baseline smoked less once they were in their forties (20.3%) than those in their 40s at baseline (26.2%). Participants with a high education level smoked less on every round of the entire study. There was an increase in participants deviating from 7 or 8 h of sleep a night during the study, from 17.1 to 29% (Figure 2D). Those in their 20s at baseline had a higher prevalence of unhealthy sleep by the time they were in their 40s (22.4%) than those in their 40s at baseline (16.3%). Low level educated participants systematically slept more often unhealthily. The proportion of individuals consuming more than 1 glass of alcohol daily increased during the study, followed by a small decrease (overall from 33.3 to 32.3%). This percentage was much higher for men (52.3 to 44.2%) than for women (16.4 to 21.8%). All 10-year generations showed this same pattern—those in their 20s at baseline continued to drink less during the entire study; of the higher-educated subjects, 38.7% drank more than 1 glass per day in the first round and 41% in the last round, for the low educated this was 29.8 and 26.9%, respectively (Figure 2E).

### Individual changes in single health-related lifestyle factors over 25 years

**Figure 3:**
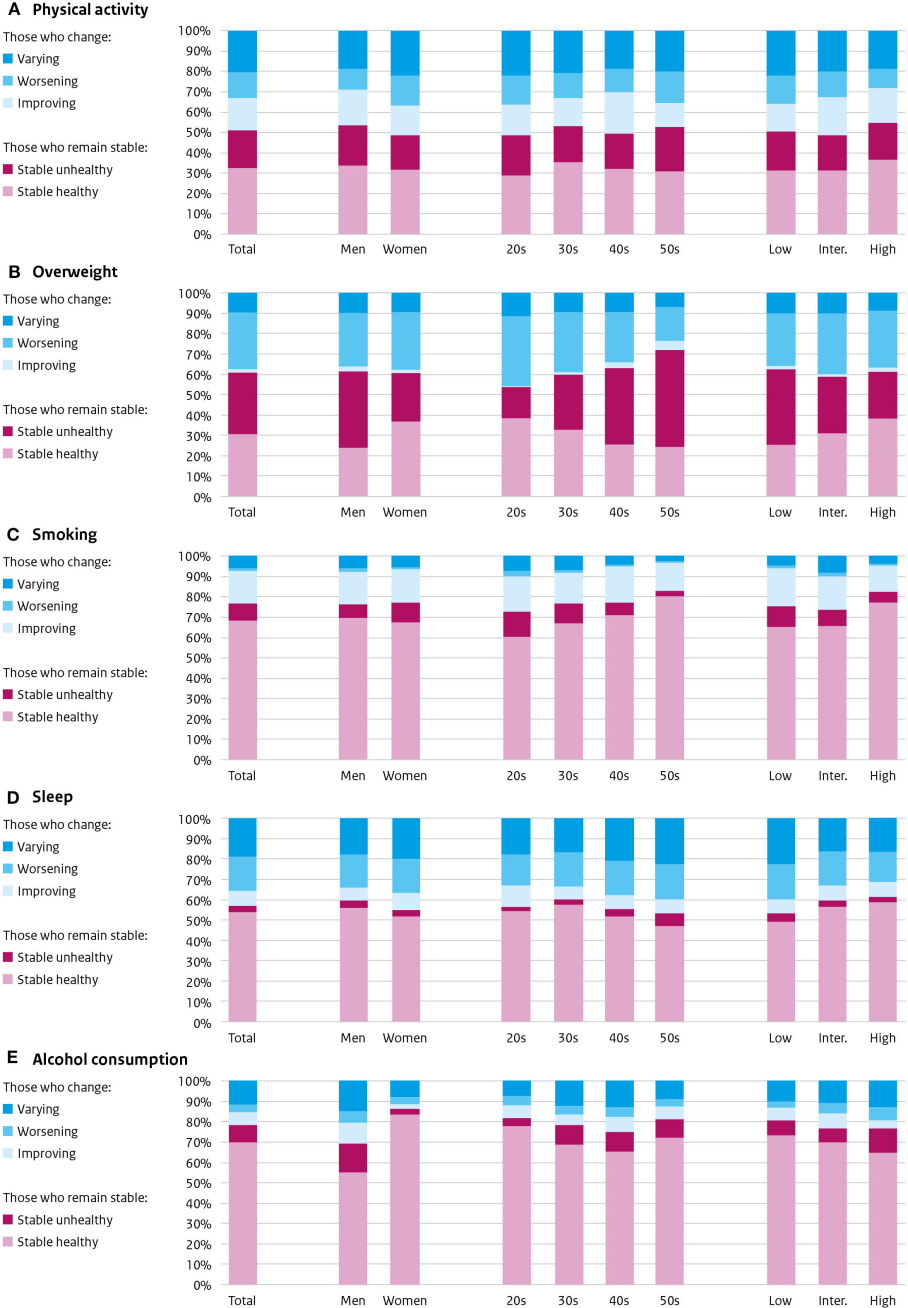
Individual changes over 25 years in **(A)** physical activity, **(B)** overweight, **(C)** smoking, **(D)** sleep, and **(E)** alcohol consumption: those who remain stable (stable healthy or stable unhealthy) and those who change (improve, worsen or vary). By sex, 10-year generation, and educational level.

Stable physical activity was found among 51% of the population (33.7% stable healthy, 18.3% stable unhealthy), and 49% changed (20.4% varied over time, 15.9% improved, 12.7% worsened) (Figure 3A). This was more or less comparable for both sexes, all 10-year generations, and all educational levels.

Stable weight was found among 61% of the participants: 30.7% stable healthy and 30.3% stable unhealthy (Figure 3B). Most of those who changed (39%) became overweight (27.5%), some varied over time (9.7%) and a small proportion improved (1.8%). Predominantly men, older generations, and lower educated were stable unhealthy.

The majority of the population showed stable smoking behavior: 76.4% (68% stable healthy non-smoker, 8.4% stable unhealthy smoker) (Figure 3C). During the study, 23.6% changed: 16.2% quitted smoking, some varied (6%), only some started (1.4%). Bigger stable healthy proportions were found among the older generations and among higher educated participants.

Sleep was found to remain stable in 56.9% of the participants: predominantly stable healthy (53.6%), a few stable unhealthy (3.3%) (Figure 3D). Those that changed their sleep duration (43.1%) were made up of 19.3% varying sleepers, 16.4% that worsened, and 7.4% that improved. Most change (any) was found in women (45.3%), low educated (47.1%), and 50s at baseline (46.6%).

Alcohol consumption did not change in 70.4:50.2% maintained a healthy consumption (stable healthy); 20.2% was stable unhealthy (Figure 3E). Those who changed their alcohol consumption (29.6%) mostly varied over time (12.8%), and some improved (9%) or worsened (7.8%). The most stable healthy were women (65.4%), those in their 20s at baseline (60.6%), and lower educated (56.9%).

### Population trends of the health-related lifestyle factors combined

**Figure 4:**
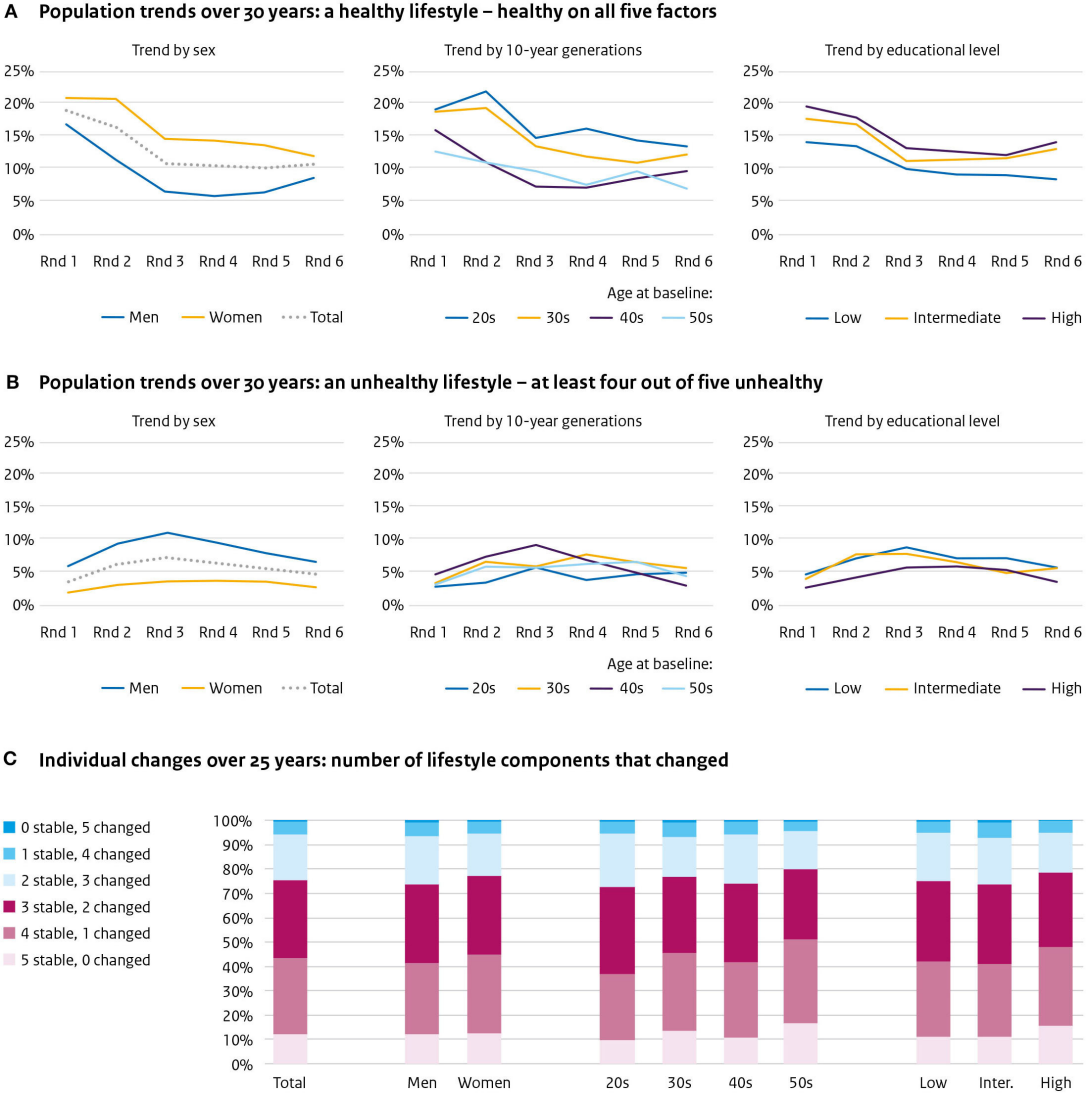
Five lifestyle factors combined. Population trends over 30 years of **(A)** a healthy lifestyle—healthy on all five factors, **(B)** an unhealthy lifestyle—at least four out of five unhealthy, and **(C)** individual changes of lifestyle over 25 years—number of components that changed.

The proportion of the population being healthy on *all five* lifestyle factors declined from 17% in the first round to 10.8% in the last round (Figure 4A). The decline was predominantly between round one (17%) and round three (10.9%). Both sexes, all 10-year generations, and educational levels showed a similar trend.

The proportion of the population unhealthy on at least four of the five factors was circa 4%, which remained more or less similar during the study (Figure 4B). For men, this was 5.7% in the first round, 10.7% in the third round, and 6.6% in the last round; for women, respectively, 1.9, 4, and 3.4%. No differences are worth noting between age or educational groups.

### Individual changes of the health-related lifestyle factors combined

In 25 years, 11% of the population did not change any of the five lifestyle factors; 29.2% changed one, 32.6% changed two, 19.8% changed three, 6.5% changed four, and 0.9% changed five (Figure 4C). The differences between the sexes and educational levels were minor. For those in their 20s at baseline, 9.4% changed none, 24.6% changed one, 36.9% changed two, 20.9% changed three, 7.1% changed four, and 1.1% changed five lifestyle factors. For those in their 50s at baseline this was 14.1, 33.2, 28.3, 19.1, 4.5, and 0.8%, respectively.

## Discussion

This study described both population trends and individual changes in the same population, emphasizing that the picture visualizing individual changes is very different from the image that emerges when populations trends are shown. In 2015, the WHO declared “Surveillance of population health and wellbeing” the first Essential Public Health Operation (23). As many population-based longitudinal studies monitor lifestyle from an individual perspective, the window they offer on such changes deserves full attention and will strengthen the insights into evolution of health and wellbeing in the population.

Our study illustrates clearly the much greater dynamics of changes in lifestyle at the individual level than can be seen in population trends. This shows better the potential for change of health-related lifestyle, either for the better or for the worse, than appears when only taking national or regional aggregate figures into account. Over a period of 25 years, the majority (60%) of the participants changed at least two of the five lifestyle factors—physical activity, overweight, smoking, sleep, and alcohol consumption—while population trends showed only a small change.

In general, our findings on population trends are in agreement with current trends in high-income countries: physical activity level remained stable, prevalence of overweight increased, smoking declined, unhealthy sleep increased, and alcohol consumption remained stable (1–5, 24). This may suggest that what we found on the individual patterns may also be similar for most high-income countries.

The findings on individual lifestyle changes are also in accordance with existing literature, although only limited comparison with literature on individual changes was possible due to differences in study populations, lifestyle measurements, and study durations. We found physical activity levels to be stable in half of our population, which is in line with findings from a recent systematic literature review on 27 longitudinal studies (follow-up time from two to 34 years) on physical activity trajectories (9). Malhotra and colleagues, who followed Americans aged 25 and above for 18 years, found that 66% of the men and 41% of the women became overweight (25), which is a greater increase than in our study (43 and 37%, respectively). A study that followed Finns for 46 years from late adolescence found smoking behavior to remain relatively stable, with especially non-smokers persisting in abstaining from smoking, in line with our data (14). A study in (female) (ex-)nurses aged 55 and above followed for 14 years found a comparable proportion of healthy sleepers (49%) as in our study (26). Nine prospective cohorts (up to 28 years follow-up) from the United Kingdom, studying adults of all ages, found alcohol consumption after 30 to have remained relatively stable, similar to our findings.

For every lifestyle factor, a considerable proportion of our participants showed multiple changes between healthy and unhealthy. Most existing studies pay little or no attention to this varying category (9, 14, 25, 26), but as it seems highly relevant for the possibilities of prevention, this category should receive more attention in studies of individual changes over time.

The sex differences presented here are minor for physical activity, smoking and sleep, but large for overweight and alcohol consumption. For both, the percentages in men are higher, and especially the categories of “stable unhealthy” for these lifestyle factors are much larger than in women. This is globally the same as reported in other studies (1–5). These sex differences are quite consistent over time—as can be seen in the population trends. Differences by generation are also seen, with as most concerning finding that those in their 40s at measurement round 4 show a higher percentage of overweight and unhealthy sleep compared to those in their 40s at measurement round 1, 20 years before. These findings extend data presented before (27), and the message is the same. There are well-known differences in the prevalence of unhealthy lifestyles between those representing different socio-economic groups in the population, often based on educational level (28). These differences are also found here: overweight, smoking and unhealthy sleep were more common in the lower educated. We did not find differences for physical activity, but unhealthy alcohol consumption was more common among the higher educated. The differences by educational level seem quit consistent over time.

The findings of this study have at least two mayor implications for areas of public health: in prevention policy and research. When considering prevention, it is important to realize that population figures do not tell the entire story. To illustrate this from our findings: monitoring of individual physical activity shows that a substantial proportion of individuals changed their activity level, while the population trend was stable. These frequent individual changes emphasize that preventive initiatives should not only target change but should also include the stimulation and support to maintain an healthy lifestyle.

Second, the findings on lifestyle (in)stability should be taken into account in research. Most studies on the role of lifestyle in the development of disease are based on a one-time measurement of lifestyle of individuals. However, lifestyles are subject to change: for most lifestyle factors, at least one-fourth of the individuals did not remain stable during our study. The one-time-measured-life-style-studies do not give a good picture of the disease risks linked to them and it is likely that these risks are underestimations of the real disease risks. Our findings emphasize that more attention should be paid to lifestyle dynamics in the population. In addition, future research may also focus more on what determines lifestyle change.

The main strength of this study is the use of 30 years of longitudinal cohort data, which allowed us to study individual changes parallel with population trends. It should be noted that our approach was entirely descriptive. We did not aim to model, for instance by developing a Markov model, the probability that an individual's lifestyle behavior would improve or worsen over the life course.

When interpreting our results, there are some limitations to be taken into account. First, we chose to use simple dichotomous indicators of the health-related lifestyle factors in order to make the analyses between the indicators comparable and to analyze multiple factors simultaneously. This simplification removes details; only the transitions around the cut-off points are described. Changes within health categories, or the degree of change, are not shown using this approach. However, we used cut-off points that are commonly used and considered meaningful in health research.

Second, the basis for evaluating most of the lifestyle factors was self-report by study participants. The validity of such self-reports can be questioned. For example, physical activity is often over-reported (9), alcohol consumption is under-reported (29), and self-reported sleep only moderately correlates with measured sleep (30). For physical activity, we took overreporting into account, and for alcohol consumption, different kinds of beverages (wine, beer, liquor) were separately assessed, which according to Freunekes et al., leads to a more realistic estimation of the level of intake (29). For some of these indicators, better (more objective) measurements are currently available, particularly measurement of sleep and physical activity by wearable devices. However, these are not available yet for a large population, and certainly not retrospectively over the last 30 years. Also, one important lifestyle factor we did not include in our analyses is food consumption. We do have (extensive) data on food consumption by means of a food frequency questionnaire in measurement rounds 2–4, but not from 1 to 6 like the other indicators.

Finally, individuals that participate in (longitudinal) population-based cohorts tend to be healthier than the general population (31). This “healthy cohort effect” might result in findings that are not one-on-one translatable to the general population. The “real” picture is expected to be more unhealthy with lower population figures and larger unhealthy patterns.

## Conclusion

Monitoring five health-related lifestyle factors over a period of 25 years in the same population from both a population and an individual perspective shows the degree to which lifestyle at the individual level is more subject to change than can be seen from in population trends. This suggests that the potential for change of health-related lifestyle, either for the better or for the worse, is greater than appears when only taking national or regional aggregate figures into account.

## Data availability statement

The datasets presented in this article are not readily available because the participants' informed consent did not include consent to public availability of the data. However, the data are available upon reasonable request, by contacting the scientific committee of the Doetinchem Cohort Study by email: Doetinchemstudie@rivm.nl. Requests to access the datasets should be directed to Doetinchemstudie@rivm.nl.

## Ethics statement

The studies involving human participants were reviewed and approved by METC of University of Utrecht. The patients/participants provided their written informed consent to participate in this study.

## Author contributions

HP, PE, and ES developed the idea for the analyses. ES and AB participated in the data analyses. HP, AB, and WV participated in the data collection. All authors participated in writing the manuscript and approved the final version.

## Conflict of interest

The authors declare that the research was conducted in the absence of any commercial or financial relationships that could be construed as a potential conflict of interest.

## Publisher's note

All claims expressed in this article are solely those of the authors and do not necessarily represent those of their affiliated organizations, or those of the publisher, the editors and the reviewers. Any product that may be evaluated in this article, or claim that may be made by its manufacturer, is not guaranteed or endorsed by the publisher.
